# Obesity and arthritis of the knee Study (OAKS): study protocol for a prospective cohort study examining the treatments and outcomes of people with obesity and severe knee arthritis

**DOI:** 10.1136/bmjopen-2025-112478

**Published:** 2026-03-12

**Authors:** Fatema G Dhaif, Nicholas Parsons, David R Ellard, Jeremy Rodrigues, Andrew Metcalfe

**Affiliations:** 1 Warwick Clinical Trials Unit, University of Warwick Warwick Medical School, Coventry, UK; 2 University Hospitals Coventry and Warwickshire NHS Trust, Coventry, UK

**Keywords:** Knee, Obesity, Protocols & guidelines, Patient Reported Outcome Measures

## Abstract

**Introduction:**

People with body mass index (BMI) ≥35 kg/m^2^ have an approximately 19-fold increased risk of undergoing total knee replacement (TKR); however, many UK integrated care boards (37%) have restrictive policies which limit access to TKR for people based on BMI. Therefore, access to both surgical and non-surgical treatments varies widely, exacerbating existing health inequalities. It remains unclear how decisions about offering TKR are made in people with severe knee osteoarthritis, which weight-loss interventions are provided in practice and how different management pathways relate to patient outcomes among individuals with high BMI.

**Methods and analysis:**

This study will recruit 400 participants with severe Kellgren-Lawrence grade four knee osteoarthritis from eight secondary care centres in England. All participants, irrespective of BMI, will provide baseline clinical data and patient-reported outcome measures (PROMs), enabling characterisation of baseline associations between BMI, knee function and body image.

A prespecified subgroup of participants with BMI ≥35 kg/m^2^ (minimum n=105) will undergo longitudinal follow-up at 6 months, 12 months and 24 months, including repeat BMI measurement, PROMs and detailed data on access to surgical and non-surgical interventions, including weight-loss strategies and TKR. For those with BMI ≥35 kg/m^2^, statistical modelling will be used to explore associations between baseline factors and longitudinal outcomes including Oxford Knee Score and weight change at 12 months (n≥105). Structural equation modelling will be used to quantify associations between BMI and knee pain/function mediated by psychosocial factors using data from all participants (n=400). A nested qualitative study of surgeons and patients will explore obstacles and preferences in the management of severe knee osteoarthritis.

**Ethics and dissemination:**

The study received ethical approval from the West of Scotland REC 5 (Ref: 24/WS/0146) on 10 October 2024. Results will be disseminated through peer-reviewed publications.

**Trial registration number:**

ISRCTN42984928.

**STRENGTHS AND LIMITATIONS OF THIS STUDY**

- Prospective observational study that includes participants from eight heterogeneous sites covering a diverse local population across England aims to provide representative sample which reflects National Health Service service variation.
- Collection of routine patient-reported outcome measures (PROMs) (Oxford Knee Score and Euro-Qol Five Dimensions) in addition to broader PROMs not routinely collected (Activity Participation Questionnaire, General Self-efficacy scale and Body-Q).
- Addition of exploratory qualitative and quantitative analyses to the main cohort study.
- Does not examine primary care practices or include those with severe arthritis and obesity who do not get referred to secondary care.
- Can identify associations but cannot measure causal relationships between treatments and outcomes.

## Introduction

 Knee osteoarthritis (OA) affects approximately 4.5 million adults over the age of 45 years in England.^1^ Severe knee OA represents a major cause of pain, disability and referral to secondary care and is managed through a combination of non-surgical interventions and, for some patients, progression to total knee replacement (TKR). The pathophysiology of OA involves mechanical factors and pro-inflammatory mediators, which are both exacerbated by obesity.^2 3^ Obesity, defined by the WHO as a body mass index (BMI) of ≥30, leads to greater loading forces in the articular cartilage which can lead to tissue damage after prolonged exposure and also leads to greater presence of pro-inflammatory mediators.^4^ As a result, people with a BMI of ≥30 kg/m^2^ have 4.6 times increased risk of developing knee OA when compared with people with a BMI of <25 kg/m^2^.^5^ The risks rise steeply with higher BMI: those with a BMI of 35–39.9 kg/m^2^ have an 18.7-fold increased risk of having a TKR. This group is therefore not only at greater clinical risk but also disproportionately affected by restrictive BMI-based thresholds for accessing surgery, with 15/41 integrated care boards imposing BMI restrictions on joint replacement eligibility in England.^6^ As the proportion of adult obesity in England has increased from 14.9% to 28% since 1993, the burden of knee OA is expected to rise further.^7 8^

Greater BMI is associated with greater pain from knee OA irrespective of radiographic severity.^9 10^ The causes of greater pain in obese individuals are multifactorial and include increased mechanical loading of the joint,^11^ a pro-inflammatory state through increased inflammatory markers such as interleukin-6 and C reactive protein^12^ and the complex reciprocal relationship between obesity, pain and depression.^13^

It remains unclear which non-surgical treatments should be offered to people with obesity and end-stage knee OA in secondary care if surgery must be delayed. Uncontrolled arthritis in this population has a substantial impact on well-being and has economic consequences. A 2021 Markov analysis has estimated over £100 000 in lost earnings in those with uncontrolled OA pain compared with those with controlled symptoms.^14^ The long-term outcomes for people who are denied a TKR due to high BMI are poorly understood, but previous work has shown that restrictive BMI policies are more likely to impact those with BMI ≥35 kg/m^2^.^15^ Furthermore, an American observational study of 125 patients denied a joint arthroplasty due to high BMI found that 80% of participants denied a joint arthroplasty failed to lose sufficient weight to subsequently be offered a joint arthroplasty, but the study had a high loss to follow-up rate of 62%.^16^

To address these evidence gaps and develop more standardised guidance for managing this population, we first need a clearer understanding of current care pathways, the treatments people receive and the factors influencing their outcomes. This protocol describes a prospective cohort study designed to characterise management pathways and outcomes in people with severe knee OA, with focused longitudinal follow-up of individuals with BMI ≥35 kg/m^2^, to inform future trials and evidence-based management strategies.

### Study aim

To examine management pathways and longitudinal outcomes in people with BMI ≥35 kg/m^2^ and severe knee OA referred to secondary care and to characterise baseline patient-reported outcomes across BMI categories.

Objectives:

1. Explore and model to what degree the interactions between baseline factors described in the prior objective, treatments received after recruitment and weight change predict knee function at 12 months after presentation to secondary care in this population.
2. Qualitatively explore patient and surgeon experiences of treatments for high BMI and knee pain.
3. Describe and quantify the relationship between underlying constructs measured by key patient-reported outcome measures for both obesity and knee-related pain and function at baseline across BMI categories.

## Methods and analysis

### Setting and participants

Participants will be recruited from eight secondary referral centres in England:

- University Hospitals Coventry & Warwickshire.
- The Princess Alexandra Hospital NHS Trust.
- Luton and Dunstable University Hospital.
- Milton Keynes University Hospital.
- University Hospitals Birmingham.
- South Tees Hospitals NHS Trust.
- Wrightington, Wigan and Leigh NHS Foundation Trust.
- Northumbria Healthcare NHS Trust.

Recruitment commenced on 17 December 2024 and completed on 19 December 2025.

Inclusion criteria

- Referral from primary care to secondary or intermediate care services for knee OA.
- Severe arthritis as assessed by the treating clinician. Defined as Kellgren-Lawrence grade four primary knee arthritis (severe) in any knee compartment on a knee radiograph (X-ray), or clinician deems arthritis severe enough to warrant a TKR.
- Aged over 18 years

Exclusion criteria

- Presence of inflammatory arthritis.
- History of intra-articular fracture causing post-traumatic arthritis.
- Previous surgery on the ipsilateral knee if implants were inserted.
- Unable or unwilling to comply with follow-up procedures.
- Unable to provide informed consent (eg, does not have capacity to consent).
- Insufficient English language skills to understand study materials and respond to questionnaires.

### Study Procedures

Orthopaedic secondary care clinic lists will be screened to identify potential participants meeting the inclusion criteria. Approaches for recruitment will occur at their clinic appointment or via contact afterwards. Informed consent will be obtained, and a baseline questionnaire administered. Baseline data from participants with BMI <35 kg/m^2^ are required in addition to the baseline data from those with BMI ≥35 kg/m^2^ to allow cross-sectional modelling of BMI-related associations for all participants. Participants will continue with NHS treatment as deemed appropriate by their clinician. Longitudinal follow-up will be intentionally restricted to participants with BMI ≥35 kg/m^2^, in whom variability in access to care, mandated weight-loss interventions and delays to surgery are most pronounced. It is anticipated that some participants will undergo surgical intervention, including TKR, during follow-up, while others will remain on non-surgical management pathways, but this will be collected as part of follow-up. Follow-up will occur at 6 months, 12 months and 24 months via telephone, post or email for participants where BMI ≥35 kg/m^2^. Weighing scales will be offered to those without access to scales at home.

### Participant identification and screening

Potential participants will be screened and identified for eligibility by the site clinical teams by inspecting lists in secondary or intermediate care clinics at each of the study recruitment sites. Potential participants will be approached via telephone or in person and invited to discuss the study. Written study information may be posted to them. Potential participants will be encouraged to contact the research team directly by telephone or email after reading the study information sheets, which will be posted to them, to indicate their interest in participating in the study. A member of the local research team will carry out the informed consent process, enrolment and baseline data collection. Height and weight data will be collected by a member of the research team in person or where documented on the participant’s electronic record within the last 2 months.

All people who meet the study entry criteria will be checked for eligibility and recorded on the monthly screening log. Screening log data for participants who meet the eligibility criteria and decline to participate will include: age, sex and ethnicity.

### Baseline data collection

The baseline questionnaire will include:

- Height and weight, which will be used to calculate BMI. This will be measured and recorded by a member of the research team or extracted from the electronic record if measured within the preceding 2 months.
- Oxford Knee Score (OKS), a patient-reported outcome measure (PROM) with 12 items, each of which is scored on an ordinal scale from 0 to 4 and totalled to give an overall assessment, that was developed for assessing outcomes after TKR.^17 18^ It has been extensively validated^19^,^21^ and its psychometric properties described previously^22^ and is the core measure used in the UK National Joint Registry (scoring 0–48; 0=worst possible score, 48=best possible score).
- OKS-Activity Participation Score (APQ) was developed as an adjunct to the OKS. The purpose of the APQ is to combat potential ceiling effect for OKS when used alone in a postoperative population^23^ (0=worst possible score, 100=best possible score).
- Body-Q, a PROM which was developed for use by people with obesity undergoing weight loss interventions. It measures four domains (appearance, eating-related concerns, health-related quality of life and experience of care). An international multidisciplinary consensus meeting identified which constructs were most important to measure and identified BODY-Q as the PROM instrument which best measured six out of eight of the most important domains.^24^ Two subscales will be used: body image and appearance-related psychosocial distress. For both subscales, 0=worst possible score, 100=best possible score (Rasch scaled scores).
- Euro-Qol-Five Dimensions-5 Levels (EQ-5D-5L) is a measure of health-related quality of life that assesses mobility, self-care, usual activities, pain/discomfort and anxiety/depression (five dimensions).^25 26^ The value set for England developed by Delvin *et al*^27^ will not be used as it was rejected by the National Institute for Health and Care Excellence (NICE).^28^ Instead, the scores will be mapped onto the 3L value set,^29^ unless the NICE guidance changes regarding this, reported as an index score −0.594 (worst score)–1 (best score) and anchored at 0 (death). A Visual Analogue Scale score (0=worst score, 100=best score) also forms part of EQ-5D-5L and provides a complementary patient-reported measure of overall health status.
- Generalised Self-Efficacy scale^30^: a questionnaire used to quantify a person’s perceived ability to cope with adversity, where 10=worst score and 40=best score.
- Hospital Anxiety and Depression Scale.^31^ A scale which quantifies depression and anxiety in medical patients. Scored from 0 to 21 for each of the anxiety and depression subscales, where 0=best score (no anxiety or depression) and 21=worst score (severe distress).

### Follow-up assessments

The follow-up questionnaire will collect data on participants’ weight, weight-loss treatments that participants have received and treatments for their knee and their outcomes.

At 6 months, 12 months and 24 months, the same PROMs collected at baseline (OKS, APQ, Body-Q, EQ-5D) will be readministered, with the addition of the Global Rating of Change Scale.^32^ This is a seven-point scale ranging from ‘no change or condition has worsened’ to ‘a great deal better’. This will provide a reference point against which changes in other PROMs can be interpreted, helping to distinguish meaningful improvement from measurement variability.

Participants will additionally be asked the following questions about previous knee treatments at each follow-up assessment: have they had a knee replacement or other knee surgery, physiotherapy and/or a steroid injection in the last 6 months and/or are they on a waiting list for knee replacement surgery.

Participants will also be asked about weight loss treatments including whether they have had weight loss surgery, taken prescribed medications for weight loss, attended an NHS-referred structured weight loss programme or dietary modification programme and/or they meet the minimum NHS-recommended exercise recommendations.

### Primary outcome and analysis timepoint

The primary outcome for this study is change in OKS from baseline to 12 months. A 12-month period has been selected as the prespecified time point for the main analysis, as it represents a clinically meaningful period in which to assess functional change and weight management interventions, while also minimising loss to follow-up.

### Long-term outcomes

Participants will be consented to long-term data linkage with the National Joint Registry (NJR) and Hospital Episode Statistics (HES).^33 34^ This will allow data linkage for subsequent surgical interventions, hospital admissions and other relevant healthcare utilisation beyond the 24-month follow-up period. The 24-month PROMs and registry linkages will therefore be analysed and reported as secondary/long-term outcomes and reported separately from the main 12-month study outcomes.

### Protocol registration and deviations

The protocol was prospectively registered on ISRCTN (ISRCTN42984928, https://doi.org/10.1186/ISRCTN42984928). The design, eligibility criteria, outcomes and planned analyses described in this manuscript are consistent with the registered protocol.

No substantive deviations from the preregistered protocol have occurred. Any future protocol deviations arising during study conduct will be reported transparently in subsequent results publications in accordance with standard reporting practice.

### Sample size calculation

Previous studies at our centre predicting PROMs in knee pathology reported R² values, the proportion of the variance in outcomes accounted for by the explanatory data (ie, regression model terms), of approximately 30%. Using a more conservative R² of 20% (effect size=0.25), a sample size of 76 was estimated as required to achieve 90% power at a 5% significance level for a cross-sectional primary analysis at 12 months. For the longitudinal analysis (baseline, 6 months and 12 months), applying a design effect of 1.1 (assuming Intraclass Correlation Coefficient=0.05) increased the required sample to 84. Accounting for an expected 20% loss to follow-up, we aim to recruit a minimum of 105 participants with BMI ≥35 kg/m^2^ for the primary analysis.

In addition to the main cohort (BMI ≥35 kg/m^2^), baseline data will be collected from a comparator group with BMI <35 kg/m^2^. The original funding application assumed a BMI distribution of ~52% with BMI ≥35 kg/m^2^ and ~48% with BMI <35 kg/m^2^. However, early recruitment data indicate the actual clinical distribution is approximately 66% BMI <35 kg/m^2^ and 33% BMI ≥35 kg/m^2^. Based on this assumed split, we plan to recruit all eligible patients consecutively, regardless of BMI category, until a total sample of 400 is achieved. Given the expected split between those under and over the BMI threshold of 35 kg/m^2^, this should ensure we recruit approximately the required number of participants (n=105) for the primary analysis in the high BMI group. This sample size is consistent with the published guidelines for the planned exploratory modelling and hypothesis generation.^35 36^

This approach ensures that our final cohort will reflect the population presenting to secondary care, rather than enforcing an artificial quota by BMI category. While the comparator group of participants with BMI <35 kg/m^2^ will not be included in the primary analyses specified in this protocol, these data will be used for exploratory modelling and hypothesis generation. The study is therefore adequately powered for the planned analyses while remaining representative of the underlying clinical population.

### Statistical analysis

Descriptive statistics will be used to summarise the demographic and questionnaire data at baseline and follow-up. Means and SD for continuous and assumed continuous variables (eg, age and PROMs) and counts and percentages for categorical variables (eg, gender). Treatments for both the knee and weight management will be categorised according to NICE OA^37^ and obesity^38^ guidelines, offered and delivered and what proportion of participants complies with each of the treatment options as defined above.

A longitudinal linear regression model will be fitted using all available longitudinal data from participants with BMI ≥35 kg/m^2^ to determine the variables which predict the primary outcome (OKS) at 12 months after first presentation to secondary care. The explanatory variables will include those data collected at follow-up time points (EQ-5D, BMI change, adherence to treatments as described above, receiving TKR, BODY-Q score). In order to assess the veracity and stability of the fitted model, sensitivity analyses and a number of standard methods will be investigated for imputing missing values (eg, multivariate imputation by chained equations using mice package in R).^39^ All analyses will be performed in R with significance set at the 5% level.^40^

### Embedded qualitative study

A purposive sample of up to 20 patients from the cohort study (stratified by age, sex and BMI) and up to 20 consultant orthopaedic knee surgeons will be invited to complete semistructured qualitative interviews. The exact sample size will be determined by maximising diversity of themes covered. Interviews will be conducted either in person or via video teleconference, according to participant preference. An interview schedule will be developed with input from patient and public involvement (PPI) partners. Interviews will be recorded and transcribed verbatim and analysed using inductive thematic analysis^41^ in NVivo V.15 (QRS International, Warrington, UK). ^41^Transcripts will be read and reread from the whole data before applying individual codes which will then be organised into themes. These will be reviewed by a senior author before the data are organised into subthemes and overarching themes as described by Braun and Clarke.^41^ Analysis will be conducted alongside data collection to assess when sufficient conceptual depth and theme diversity have been achieved. Recruitment will cease once additional interviews no longer contribute meaningfully to developing or expanding the thematic framework.

## Discussion

This prospective cohort study will describe the baseline characteristics and PROMs of people with obesity and severe knee OA and explore whether these predict pain, function and weight change at 12 months. It will address a critical evidence gap in the non-surgical management of this population, who often face BMI-based restrictions to TKR by commissioners, individual hospitals or individual surgeons.

The inclusion criteria of BMI ≥35 kg/m^2^ target patients at the highest risk of poor outcomes and those most affected by commissioning restrictions, allowing us to explore the most clinically complex and underserved group. The strengths of this study include its multi-centre design, capturing practice across England, which will enhance the generalisability across the NHS. The follow-up period of 12 months will provide insight into both short-term and medium-term outcomes. In addition, participants have consented to long-term outcome collection to 24 months and through linkage with the NJR and HES, allowing ongoing monitoring of surgical interventions and healthcare utilisation beyond the study follow-up period. Long-term outcomes will be reported after completion of the main analysis in a separate publication. The use of validated PROMs alongside detailed treatment data will enable a nuanced understanding of patient journeys and inform modelling of predictors of functional outcomes.

The limitations of this study are that it will not be able to establish causal links between treatments and outcomes due to its observational design and the heterogeneity of treatment availability across sites. In addition, this study will not capture data on people with severe OA and obesity who are managed exclusively in primary care and are never referred to secondary care, limiting generalisability to this broader population. Anthropometric measures such as waist or knee circumference are not being measured, which will restrict the exploration of mechanistic or prognostic relationships.

The findings from this study will help define which non-surgical strategies are currently used in routine practice and how patient-reported outcomes vary following these interventions. By examining associations between baseline patient characteristics, management pathways and subsequent outcome, this study will provide insight into which factors are associated with better or worse pain, function and weight-related outcomes over time. This evidence base will be critical for shaping future national guidelines aimed at reducing variation in care and supporting shared decision-making for people with obesity and knee OA. Future analyses may include advanced modelling techniques (eg, structural equation modelling) to explore relationships between baseline psychosocial, clinical and outcome variables, which will be piloted and refined as part of the wider project. These analyses form part of the overall study plan but will be reported separately from the main cohort results. Ultimately, the inter-related work packages in this study will deliver a substantial increase in our understanding of the experience and treatment of people with obesity and severe knee OA, particularly those who may not be able to access surgery, a common yet poorly understood challenge in current practice.

### Patient and public involvement

Patient and public involvement began at the earliest stages of developing this research proposal. Three PPI partners with lived experience of knee OA, weight loss interventions, TKR (including revision surgery) and obesity were recruited to contribute to the design and development of the study. They informed the research questions, selection of outcome measures and the recruitment and follow-up processes, ensuring these reflected patient priorities and were feasible and acceptable to participants.

These PPI partners joined the project steering group and participated in regular meetings throughout the pre-study phase. They reviewed study materials (including participant information sheets and questionnaires) for clarity, accessibility and burden, and provided feedback to optimise the participant experience. They also advised on strategies to improve recruitment and retention.

During the conduct of the study, PPI partners will continue to provide input on ongoing recruitment challenges, support the interpretation of findings and help prioritise which results are most important to share with patients and the wider community. They will also contribute to the development of dissemination plans, including the format and content of study results to ensure these are accessible and meaningful to patient communities. We will use the PPI Impact Assessment Framework^42^ to document the PPI process.

### Ethics and dissemination

The study received ethical approval from the West of Scotland REC 5 (Ref: 24/WS/0146) on 10 October 2024. Results will be disseminated through peer-reviewed publications.
