# Supplementary material for: Obesity and arthritis of the knee Study (OAKS): study protocol for a prospective cohort study examining the treatments and outcomes of people with obesity and severe knee arthritis
Source: BMJ Open. 2026 Mar 12;16(3):e112478. doi: 10.1136/bmjopen-2025-112478 (PMC12983940; doi:10.1136/bmjopen-2025-112478)
Supplement: Supplementary file 1 [file bmjopen-2025-112478.reviewer_comments.pdf]

PEER REVIEW HISTORY

BMJ Open publishes all reviews undertaken for accepted manuscripts. Reviewers are asked to complete a checklist review form and are provided with free text boxes to elaborate on their assessment. These free text comments are reproduced below.

ARTICLE DETAILS

Title (Provisional)

Obesity and Arthritis of the Knee Study (OAKS): study protocol for a prospective cohort study examining the treatments and outcomes of people with obesity and severe knee arthritis

Authors

Dhaif, Fatema G; Parsons, Nicholas; Ellard, David R; Rodrigues, Jeremy; Metcalfe, Andrew

---

VERSION 1 - REVIEW

**Reviewer**	**1**
**Name**	**Runhaar, Jos**
**Affiliation**	**Erasmus University Medical Center Rotterdam**
**Date**	**08-Jan-2026**
**COI**	**None**

This study protocol describes a prospective cohort study examining the treatments and outcomes of people with obesity and severe knee arthritis (OAKS). The protocol is generally well-written, but there are some issues with the reporting.

In order of appearance:

Abstract

“many UK clinical commissioning groups (68%) have restrictive policies which limit access to TKR […]”. The number of 68% is rather confusing as it isn’t explained (nor in abstract, nor in the manuscript) how the authors obtained this estimation.

ISRCTN 42984928: Although pre-registration is appreciated and deviations of pre-defined plans could be acceptable, transparency on each protocol deviation is crucial but currently lacking from the manuscript.

After presenting the study as a cohort of 400 individuals with sever knee OA in the abstract, it seems odd to focus the Introduction towards (severe) obese individuals only. It seems (parts of) the Introduction, Study aim, Methods and analysis, and Discussion are written for a cohort of ≥105 individuals with sever OA and BMI ≥35.

“This prospective cohort study will describe the baseline characteristics and PROMs of people with obesity and severe knee OA and explore whether these predict pain, function and weight change at 12 months. It will address a critical evidence gap in the non-surgical management of this population […]”. Here again, the authors narrow down their aims more strictly than what the target population represents. If the authors indeed “recruit all eligible patients consecutively, regardless of BMI category”, those with BMI <35 will not all be obese, but also will include normal-weight individuals. Moreover, it seems unlikely that when enrolling individuals with “Kellgren-Lawrence grade 4 primary knee arthritis (severe) in any knee compartment on a knee radiograph (x-ray), or clinician deems arthritis severe enough to warrant a TKR” that the interventions these individuals will undergo in the following 12 months be solely non-surgical.

Upon acknowledging that the proposed study “will not be able to establish causal links between treatments and outcomes due to its observational design” and based on the presented analyses, it is unclear how “the findings from this study will help define […] how effective they [non-surgical strategies] are in practice, and which patient factors influence treatment response”.

As indicated in the instruction for reviewing protocol papers, the dates of the study should be included in the manuscript.

VERSION 1 - AUTHOR RESPONSE

We thank the Editor and Reviewers for their careful and constructive assessment of our manuscript. We have revised the manuscript in response to all comments and believe the changes have improved the clarity and coherence of the protocol. Below, we provide a detailed point-by-point response outlining how each comment has been addressed. All changes are reflected in the revised manuscript and highlighted in the marked copy.

Comment 1:
Abstract
“many UK clinical commissioning groups (68%) have restrictive policies which limit access to TKR […]”. The number of 68% is rather confusing as it isn’t explained (nor in abstract, nor in the manuscript) how the authors obtained this estimation.

Response 1:
We thank the reviewer for their comment and agree that the origin and context of this estimate were insufficiently explained. The original figure was derived from a 2023 review of Clinical Commissioning Group (CCG) policies, which reported that 67.9% (72/106) of CCGs had BMI-related policies affecting access to hip or knee arthroplasty, with many introducing additional waiting times or restrictions based on BMI thresholds. (McLaughlin, J., Elsey, J., Kipping, R. *et al.* Access to hip and knee arthroplasty in England: commissioners’ policies for body mass index and smoking status and implications for integrated care systems. *BMC Health Serv Res* **23**, 77 (2023). https://doi.org/10.1186/s12913-022-08999-9)

Since the restructuring of commissioning bodies in England and the transition from CCGs to Integrated Care Boards (ICBs), more recent evidence indicates substantial variability in current policy approaches with 15/41 (37%) of ICBs having a restrictive policy in place. We have therefore revised the abstract and introduction with this updated figure and referenced it appropriately. (Ofosu K, Whale K, McLaughlin J. Persistent use of body mass index policies as a barrier to surgery: Prevalence and analysis of policies across England in 2025. Journal of Health Services Research & Policy. 2025;0(0). doi:10.1177/13558196251405207)

Manuscript changes
Abstract, Introduction (line 26 and 119), reference number 6

Comment 2:
ISRCTN 42984928: Although pre-registration is appreciated and deviations of pre-defined plans could be acceptable, transparency on each protocol deviation is crucial but currently lacking from the manuscript.

Response 2:
We thank the reviewer for highlighting the importance of transparency in relation to protocol registration. This manuscript describes the planned study protocol, and at the time of submission no substantive deviations from the preregistered protocol had occurred. The study design, eligibility criteria, outcomes, and planned analyses are consistent with the ISRCTN registration.
We have clarified this explicitly in the Methods section. Any deviations from the protocol that arise during the conduct of the study will be reported transparently in subsequent results publications, in line with standard reporting practice.

Manuscript changes:
Methods, line 355

Comment 3:
After presenting the study as a cohort of 400 individuals with sever knee OA in the abstract, it seems odd to focus the Introduction towards (severe) obese individuals only. It seems (parts of) the Introduction, Study aim, Methods and analysis, and Discussion are written for a cohort of ≥105 individuals with sever OA and BMI ≥35.

Response 3:
We thank the reviewer for highlighting this issue and agree that, in the original manuscript, the emphasis on individuals with BMI ≥35 was not sufficiently contextualised, which may have given the impression that the study population was restricted to this subgroup. We have revised the manuscript throughout to ensure consistency between the stated cohort and the analytic focus.

Specifically, the Introduction has been revised to establish severe knee osteoarthritis as the primary clinical condition under study, with BMI framed as an important modifier examined both as a continuous variable at baseline and as a policy-relevant subgroup at higher thresholds. The study aim has been rewritten to explicitly reflect the two-tier design, distinguishing baseline analyses conducted across all BMI

categories from longitudinal analyses focused on a pre-specified BMI ≥35 subgroup. The Methods section now clearly states the purpose of recruiting participants across BMI categories and the rationale for selective longitudinal follow-up, and the Discussion has been revised to avoid language that implied the cohort comprised only individuals with BMI ≥35.

Comment 4:
"This prospective cohort study will describe the baseline characteristics and PROMs of people with obesity and severe knee OA and explore whether these predict pain, function and weight change at 12 months. It will address a critical evidence gap in the non-surgical management of this population […]". Here again, the authors narrow down their aims more strictly than what the target population represents. If the authors indeed "recruit all eligible patients consecutively, regardless of BMI category", those with BMI <35 will not all be obese, but also will include normal-weight individuals. Moreover, it seems unlikely that when enrolling individuals with "Kellgren-Lawrence grade 4 primary knee arthritis (severe) in any knee compartment on a knee radiograph (x-ray), or clinician deems arthritis severe enough to warrant a TKR" that the interventions these individuals will undergo in the following 12 months be solely non-surgical.

Response 4:
We thank the reviewer for this comment and agree that the original wording of the aims and abstract was overly restrictive. The cohort recruits all eligible individuals with severe knee osteoarthritis consecutively, regardless of BMI, in order to support robust baseline modelling of the relationship between BMI, knee-related outcomes, and body image. Participants with BMI <35 contribute baseline data only and are not followed longitudinally.

Longitudinal follow-up is intentionally restricted to a pre-specified subgroup of participants with BMI ≥35. This group is the primary population of interest because they are more likely to experience heterogeneity in management decisions, including progression to surgery, delays to surgery, or non-surgical management pathways influenced by BMI-related policies. Importantly, participants are followed prospectively irrespective of eventual treatment received, and both surgical and non-surgical interventions are anticipated and captured during follow-up.

We agree with the reviewer that, given the severity of knee osteoarthritis at enrolment, it is expected that some participants will undergo surgical intervention, including total knee replacement, during the follow-up period, while others will not. The study is observational in nature and is designed to capture the range of management pathways experienced in routine care rather than to assume exclusively non-surgical management. We have revised the abstract and study aims to clarify that the study is observational in nature and that the focus is on describing real-world management pathways and associated outcomes rather than restricting analyses to non-surgical care.

Manuscript changes:
Abstract (Methods and analysis section).

Comment 5:
Upon acknowledging that the proposed study "will not be able to establish causal links between treatments and outcomes due to its observational design" and based on the presented analyses, it is unclear how "the findings from this study will help define […] how effective they [non-surgical strategies] are in practice, and which patient factors influence treatment response".

Response 5
We thank the reviewer for this comment and agree that the original wording could be interpreted as implying causal inference beyond the scope of the study design. We have revised the Discussion to clarify that the study is observational and will describe patterns of care and associations between management strategies, patient characteristics, and subsequent outcomes, rather than evaluating causal effectiveness or treatment response.

We now explicitly frame the contribution of the study as informing understanding of real-world management pathways and outcome variation, and as providing evidence to support future interventional studies and guideline development.

Manuscript changes:
Discussion section line 468.

Comment 6:
As indicated in the instruction for reviewing protocol papers, the dates of the study should be included in the manuscript.

Response 6:
Recruitment commenced on 17 December 2024 and completed on 19 December 2025.

Manuscript changes:
Line 193 (methods and analysis section)
